# Living alone and risk of dementia, cognitive decline, and institutionalization in the MEMENTO cohort

**DOI:** 10.3389/fragi.2026.1782176

**Published:** 2026-04-28

**Authors:** Dominique Huvent-Grelle, Jean Baptiste Beuscart, Audrey Hubert, Vincent Bouteloup, Philippe Amouyel, François Puisieux, Aghiles Hamroun, Estelle Aymes

**Affiliations:** 1 Department of Geriatrics, Lille University Hospital, Lille, France; 2 ULR2694 – METRICS: Health Technology and Medical Practice Assessment, Lille University Hospital, Lille, France; 3 Public Health Department, Lille University Hospital, Lille, France; 4 Inserm UMR1167 RID-AGE, Institut Pasteur de Lille, Lille, France; 5 Bordeaux Population Health, University of Bordeaux, Inserm, UMR1219, Bordeaux, France; 6 CIC1401 EC, Pôle Santé Publique, CHU de Bordeaux, Bordeaux, France

**Keywords:** dementia, institutialization, living alone, MEMENTO cohort, mild cognitive impairment

## Abstract

**Introduction:**

Alzheimer’s disease and related dementias (ADRD) represent a public health challenge, with prevention strategies focusing on modifiable risk factors such as isolation. Living alone is used as a proxy for social isolation, although its relationship with ADRD outcomes remains unclear, partly due to the distinction between objective isolation and subjective loneliness. This study examined the association between living alone and the risk of dementia, cognitive decline, and institutionalization in the MEMENTO cohort, a French clinic-based study of individuals with cognitive complaints or mild cognitive impairment.

**Methods:**

Living alone at baseline was the main exposure. Perceived isolation was assessed using self-reported measures. Outcomes included incident dementia, institutionalization; and trajectories of Mini-Mental State Examination (MMSE) scores over a 5-year median follow-up. Cause-specific Cox models accounting for competing risks were used for dementia and institutionalization, and linear mixed models for MMSE trajectories.

**Results:**

Among 2,269 participants (median age 71.5 years, 62% women, median MMSE 28), 30.7% lived alone and 6.5% reported perceived isolation. At 60 months, estimated cumulative incidences were 15% for dementia, 1.0% for institutionalization and 3.6% for death. Living alone was not associated with incident dementia (HR = 0.88 [95%CI: 0.67–1.16], p = 0.38), or cognitive decline. In contrast, it was associated with a higher risk of institutionalization (HR = 3.21 [95%CI: 1.09–9.48], p = 0.03).

**Discussion:**

Living alone was not linked to dementia risk or cognitive decline, but was associated with a higher risk of institutionalization. This finding may indicate that living alone captures vulnerability related to reduced day-to-day support rather than cognitive decline itself.

## 1 Introduction

Alzheimer’s disease and related dementias (ADRD) affect over 55 million people worldwide, a number projected to reach 152 million by 2050 (Nichols et al., 2022), posing major public health and societal challenges and placing immense strain on healthcare systems and societies worldwide (Takizawa et al., 2014).

Given the lack of curative treatments, prevention strategies targeting modifiable risk factors are crucial. Among these, social isolation, accounting for roughly 5% of the population-attributable fraction (Livingston et al., 2024; Gil-Peinado et al., 2023), has gained attention.

Living alone is frequently considered a pragmatic but imperfect indicator of social isolation in epidemiological research (Gil-Peinado et al., 2023). One difficulty lies in the conceptual confusion between living alone, an objective lack of social contact, and loneliness, a subjective emotional experience (Sutin et al., 2020; Holt-Lunstad et al., 2015). Additionally, methodological heterogeneity across studies, including differences in how isolation is defined and measured, complicates comparisons.

Meta-analyses have shown associations between social isolation and both cognitive decline and increased dementia risk (Lara et al., 2019; Harrington et al., 2023). However, the nature of this relationship remains incompletely understood (Sherman et al., 2024; Kuiper et al., 2016). Longitudinal data suggest that dementia and social isolation may evolve in parallel, pointing to complex, possibly bidirectional dynamics (Xiang et al., 2021). Recent findings have challenged previous assumptions. For instance, Karakose et al. (Karakose et al., 2025) found that marital status, a factor once thought to be protective against dementia, showed inconsistent associations with dementia risk.

This study primarily examined the association between living alone and the risk of dementia, cognitive decline, and institutionalization in the MEMENTO cohort, while also considering perceived isolation as a distinct subjective social measure.

## 2 Materials and methods

### 2.1 Study design

Data were retrieved from the MEMENTO cohort, a clinic-based cohort of participants consulting in French memory clinics for isolated cognitive complaints or recently diagnosed mild cognitive impairment (MCI) (Dufouil et al., 2017). From April 2011 to June 2014, this prospective multicenter study included patients from 26 of France’s 28 university-hospital-based memory clinics (Morris, 1993) with very mild to mild cognitive impairment, regardless of their age, or isolated subjective cognitive complaint (if aged≥60 years). Cognitive impairment was defined as a SD decrement in one or more domains and Clinical Dementia Rating (CDR) (Morris, 1993) score ≤0.5 without dementia. Occurrence of dementia was evaluated during each on-site visit by the clinician’s center. All dementia cases were adjudicated by an expert panel.

Our analysis included participants living at home at their enrolment with data on living arrangements, excluding those in institutional or communal settings such as care facilities or religious communities.

### 2.2 Ethics and approvals

The MEMENTO cohort adhered to the Declaration of Helsinki and was approved by the institutional review board (reference: 2010-A01394-35). All participants provided written consent. Our study was reviewed and approved by the MEMENTO cohort data access committee.

### 2.3 Data collection

A standardized evaluation form was used in the MEMENTO study for follow-up every 6 months for 5 years (Dufouil et al., 2017). Among the comprehensive clinical, neurological and cognitive evaluations, our analysis used socio-demographic, social, clinical, and follow-up data. The instrumental activities of daily living (IADL) score was corrected according to Dufournet et al. (2021), by averaging the answered domains (range 1–4, with higher scores indicating greater dependence).

### 2.4 Living alone, perceived isolation, and outcomes

The primary exposure was living alone, defined according to residential status at baseline. Perceived isolation was considered a separate subjective social measure, assessed at baseline through a self-reported question: “Is there someone the participant feels especially close to, with whom they can share their problems and from whom they receive comfort?” A negative response was used to identify participants experiencing perceived isolation. The primary outcome was dementia onset, defined as the time from inclusion to diagnosis. Observations were censored at institutionalization or death. The secondary outcome was institutionalization before dementia diagnosis, defined as the time from inclusion to the date of admission to a medicalized long-term care facility; censored at dementia diagnosis or death. Participants who experienced none of these events were censored at their last follow-up visit.

Cognitive function was assessed with the Mini-Mental State Examination (MMSE) at each follow-up visit; scores were normalized (Philipps et al., 2014) and rescaled to the original 0–30 range for analytic purposes.

### 2.5 Statistical analysis

Categorical variables were reported as frequency (percentage), quantitative ones as mean with standard deviation (SD) or median with interquartile range (IQR). Normality was assessed graphically. Patient and disease characteristics were compared according to living arrangement status using Chi-square or Fisher’s exact test for categorical data, and Student’s t-test or Wilcoxon test for continuous variables.

Cumulative incidences of dementia were estimated with Aalen-Johansen method (Aalen and Johansen, 1978), accounting for competing events (institutionalization and death), and compared using the Gray method (Gray, 1988).

Both living alone and perceived isolation were simultaneously included in the cause-specific Cox models, with each variable mutually adjusted for the other. The Hazard Ratio (HR) and 95% Confidence Interval (95%CI) for living alone addresses the primary research question; the HR (95%CI) for perceived isolation, reported separately in the Results, addresses the secondary question regarding the subjective dimension of social isolation. These models were also adjusted for potential confounders identified in the literature, including age at inclusion, sex, last occupation, smoking status, alcohol consumption, home care, receipt of financial assistance, IADL, body mass index (BMI), history of diabetes or antidiabetic treatment at baseline, depression history, nutritional status (using Mini Nutritional Assessment), CDR score and time from first symptoms to inclusion (Livingston et al., 2024; Wang et al., 2024). Cardiovascular disease history and hypertension treatment were also included due to their known confounding effect (Cené et al., 2022; Freak-Poli et al., 2021; Kauko et al., 2024).

Proportional hazard assumptions were checked using Schoenfeld residuals. Missing data were managed by multiple imputation with chained Equation 20 iterations, 20 datasets).

To investigate the association between living alone and cognitive decline over time, we conducted a multivariable linear mixed-effects model with MMSE scores as the outcome. The model included fixed effects for time (visits), living alone, their interaction term (visit × living alone), and all previously described confounding variables. Random effects for intercept and slope were included to account for inter-individual variability. The p-value for the interaction term was extracted to assess whether the trajectory of MMSE scores differed according to baseline living arrangement. For reporting, we focused on the estimated β coefficient for time (visit), both overall and stratified by living arrangement (living alone vs. not living alone).

A 5% significance threshold was applied. Analyses were done with R software version 4.3.3 (R Core Team, 2024).

## 3 Results

### 3.1 Study population

**FIGURE 1:**
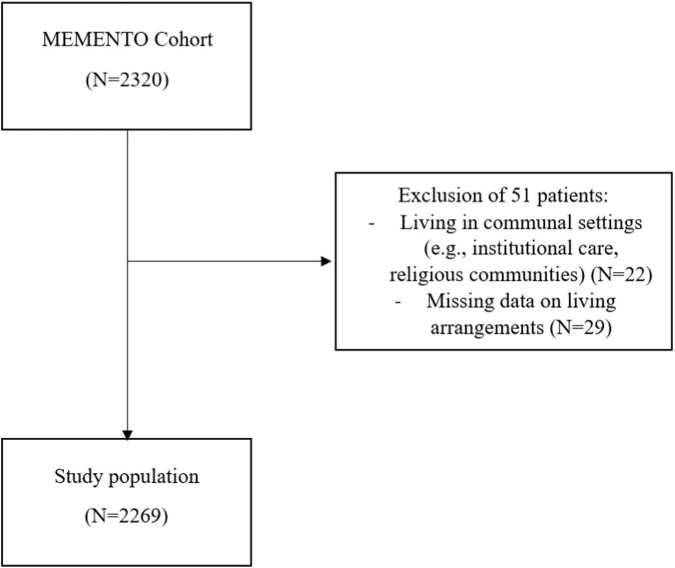
Study flowchart.

Among the 2,269 included participants (Figure 1), median age was 71.6 years [IQR 65.5.77.1], 61.7% were women (n = 1,399; Table 1) and median MMSE score at inclusion was 28 [IQR 27.29]. At baseline, 30.7% lived alone (n = 696) and 6.5% reported perceived isolation (n = 148).

**TABLE 1:** Characteristics of the study population, according to living arrangement at baseline.

​	Total	Not living alone	Living alone	p-value	Missing data
	N = 2,269	N = 1,573 (69.3%)	N = 696 (30.7%)		
Sociodemographic characteristics					
Age at inclusion (years), median [IQR]	71.5 [65.5, 77.1]	70.9 [64.6, 76.6]	73.4 [67.8, 78.3]	<0.001	-
Female	1,399 (61.7%)	810 (51.5%)	589 (84.6%)	<0.001	-
Educational level	​	​	​	0.23	-
No formal education or primary school	276 (12.2%)	180 (11.4%)	96 (13.8%)	​	​
Lower secondary school	515 (22.7%)	357 (22.7%)	158 (22.7%)	​	​
Upper secondary school	541 (23.8%)	368 (23.4%)	173 (24.9%)	​	​
Higher education	937 (41.3%)	668 (42.5%)	269 (38.6%)	​	​
Professional situation	​	​	​	0.001	<0.1%
Not working or retired	2006 (88.5%)	1,367 (87.0%)	639 (91.8%)	​	​
Working	262 (11.5%)	205 (13.0%)	57 (8.2%)	​	​
Last occupation	​	​	​	0.001	12.1%
Farmer	26 (1.3%)	16 (1.2%)	10 (1.6%)	​	​
Craftsperson	58 (2.9%)	46 (3.4%)	12 (1.9%)	​	​
Merchant or business owner	124 (6.2%)	90 (6.6%)	34 (5.4%)	​	​
Executives and senior intellectual professions	603 (30.2%)	437 (32.1%)	166 (26.3%)	​	​
Intermediate professions	394 (19.8%)	259 (19.0%)	135 (21.4%)	​	​
Employee	504 (25.3%)	311 (22.8%)	193 (30.6%)	​	​
Laborer	124 (6.2%)	93 (6.8%)	31 (4.9%)	​	​
Other	161 (8.1%)	111 (8.1%)	50 (7.9%)	​	​
Age at first signs (years), median [IQR]	66.7 [59.9, 73.3]	66.0 [59.2, 72.8]	68.2 [61.5, 74.3]	<0.001	6.1%
Family history of dementia	953 (42.3%)	673 (43.1%)	280 (40.3%)	0.24	0.6%
Lifestyle and social factors					
Smoking status	​	​	​	<0.001	0.1%
Non-smoker	1,328 (58.6%)	870 (55.4%)	458 (65.8%)	​	​
Active smoker or former smoker	939 (41.4%)	701 (44.6%)	238 (34.2%)	​	​
Alcohol consumption	​	​	​	<0.001	1.3%
None	750 (33.5%)	458 (29.5%)	292 (42.5%)	​	​
<7 times/week	801 (35.8%)	560 (36.1%)	241 (35.1%)	​	​
≥7 times/week	688 (30.7%)	534 (34.4%)	154 (22.4%)	​	​
Perceived isolation	144 (6.5%)	85 (5.5%)	59 (8.6%)	0.01	1.3%
Home care	629 (27.7%)	413 (26.3%)	216 (31.0%)	0.02	<0.1%
Financial assistance	28 (1.2%)	15 (1.0%)	13 (1.9%)	0.11	0.4%
IADL, median [IQR]^1^	9.0 [9.0, 10.0]	9.0 [9.0, 10.0]	9.00 [9.0, 10.0]	0.44	4.8%
Nutritional status poor or at risk	130 (6.1%)	71 (4.9%)	59 (9.0%)	<0.001	6.8%
BMI (kg/m^2^), median [IQR]	25.0 [22.6, 27.8]	25.2 [22.8, 28.0]	24.4 [22.1, 27.5]	0.001	2.0%
Medical history and comorbidities					
Long-term illness	615 (27.1%)	425 (27.1%)	190 (27.3%)	0.94	0.1%
Treated hypertension at inclusion	788 (34.7%)	548 (34.8%)	240 (34.5%)	0.91	-
Treated dyslipidemia at inclusion	642 (28.3%)	447 (28.4%)	195 (28.0%)	0.89	-
Diabetes	256 (11.3%)	190 (12.1%)	66 (9.5%)	0.08	-
Cardiovascular disease	307 (13.5%)	214 (13.6%)	93 (13.4%)	0.93	-
Cerebro-cardiovascular disease	1,115 (49.2%)	772 (49.1%)	343 (49.3%)	0.98	<0.1%
Depression	840 (37.0%)	516 (32.8%)	324 (46.6%)	<0.001	<0.1%
Cognitive and clinical assessment					
MMSE score, median [IQR]^2^	28 [27,29]	28 [27,29]	28 [27,29]	0.22	<0.1%
Clinical dementia rating score	​	​	​	0.02	0.4%
0	913 (40.4%)	607 (38.8%)	306 (44.0%)	​	​
0.5	1,346 (59.6%)	959 (61.2%)	387 (55.6%)	​	​
Time between first symptoms and inclusion (years), median [IQR]	3.0 [1.6, 5.7]	3.0 [1.6, 5.4]	3.1 [1.6, 6.8]	0.13	6.1%
Anti-alzheimer treatment^1^	30 (1.3%)	19 (1.2%)	11 (1.6%)	0.61	0.1%
Neuroimaging and follow-up					
MRI performed at inclusion	2,151 (94.8%)	1,500 (95.4%)	651 (93.5%)	0.09	-
Total number of follow-up visits, median [IQR]	9 [6,10]	9 [6,10]	9 [5,10]	0.15	-

IADL, instrumental activities of daily living; BMI, body mass index; MMSE, mini mental status examination.

1 Acetylcholinesterase inhibitors or NMDA, receptor antagonists.

Individuals living alone were, on average, older at symptoms onset and inclusion, more likely female, nonsmokers, nondrinkers, with higher prevalence of depression history and higher IADL scores than those not living alone.

### 3.2 Incidence of dementia, institutionalization, and death

Median follow-up was 5 years [IQR 4.1.5.1]; 318 individuals were diagnosed with dementia before institutionalization after a median of 2.3 years [IQR 1.1.3.7]. Median time to institutionalization before dementia was 3.3 years [IQR 2.2–4.0].

**FIGURE 2:**
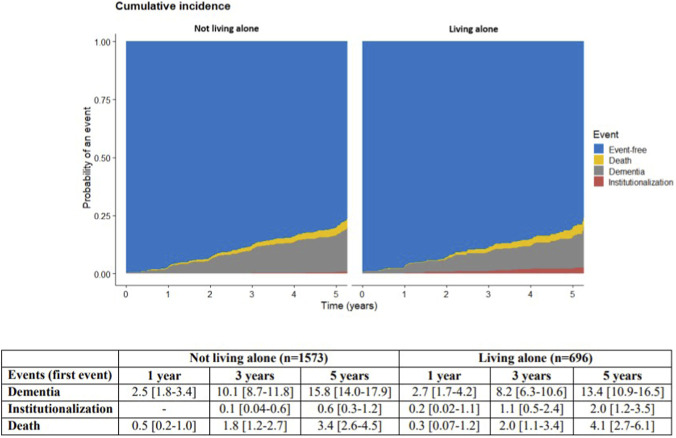
Cumulative incidence of institutionalization, dementia, and death according to residential status.

At 60 months, unadjusted cumulative incidences were 15.1% [95% CI: 13.6–16.8] for dementia, 1.0% [95% CI: 0.6–1.6] for institutionalization, and 3.6% [95% CI: 2.9–4.7] for death. Among those not living alone, incidences were 15.8% [95% CI: 14.0–17.9], 0.6% [95% CI: 0.3–1.2], and 3.4% [95% CI: 2.6–4.5]; for those living alone, they were 13.4% [95% CI: 10.9–16.5], 2.0% [95% CI: 1.2–3.5], and 4.1% [95% CI: 2.7–6.1], for dementia, institutionalization and death, respectively (Figure 2). Institutionalization incidence was significantly higher for individuals living alone (Gray’s test, p = 0.002). No significant difference was found for dementia and death (Gray’s test, p = 0.10 and p = 0.68, respectively).

### 3.3 Risk of dementia or institutionalization

In the main adjusted models, living alone was associated with a non-significant lower hazard of dementia (HR = 0.88 [95%CI: 0.67–1.16], p = 0.38), but with a significantly higher hazard of institutionalization (HR = 3.21 [95%CI: 1.09–9.48], p = 0.03). In the same adjusted model, perceived isolation was not significantly associated with incident dementia (HR = 1.06 [95%CI: 0.67.1.69], p = 0.80).

### 3.4 Association between living alone and MMSE score trajectory

At baseline, median MMSE score was 28 [IQR = 27,29] in both groups (p = 0.22; Table 1). At 5-year, MMSE scores were available for 65% of participants (N = 1,474). Descriptive MMSE distributions across follow-up visits are shown in Table 2 and did not differ significantly between groups.

**TABLE 2:** MMSE scores distribution during annual follow-up, according to living arrangement at baseline.

​	Total	Not living alone	Living alone	p-value	Missing data
	N = 2,269	N = 1,573 (69.3%)	N = 696 (30.7%)		
MMSE score across visits					
Baseline (N = 2,269)					
Median [Q1, Q3]	28.0 [27.0, 29.0]	28.0 [27.0, 29.0]	28.0 [27.0, 29.0]	0.22	0.1%
Mean (SD)	27.9 (1.93)	28.0 (1.87)	27.8 (2.04)	​	​
1-year visit (N = 2088)					
Median [Q1, Q3]	29.0 [27.0, 29.3]	29.0 [27.0, 30.0]	29.0 [27.0, 29.0]	0.56	6.5%
Mean (SD)	27.9 (2.34)	27.9 (2.35)	27.8 (2.31)	​	​
2-year visit (N = 1951)					
Median [Q1, Q3]	29.0 [27.0, 30.0]	29.0 [27.0, 30.0]	29.0 [27.0, 30.0]	0.94	6.2%
Mean (SD)	27.7 (2.81)	27.7 (2.81)	27.7 (2.82)	​	​
3-year visit (N = 1796)					
Median [Q1, Q3]	29.0 [27.0, 30.0]	29.0 [27.0, 30.0]	29.0 [27.0, 30.0]	0.25	8.2%
Mean (SD)	27.5 (3.11)	27.5 (3.20)	27.7 (2.87)	​	​
4-year visit (N = 1,670)					
Median [Q1, Q3]	29.0 [27.0, 30.0]	29.0 [27.0, 30.0]	29.0 [27.0, 30.0]	0.84	5.8%
Mean (SD)	27.3 (3.82)	27.2 (4.09)	27.6 (3.06)	​	​
5-year visit (N = 1,474)					
Median [Q1, Q3]	29.0 [27.0, 30.0]	29.0 [26.0, 30.0]	29.0 [27.0, 30.0]	0.38	1.7%
Mean (SD)	27.2 (4.07)	27.0 (4.34)	27.5 (3.31)	​	​

MMSE, mini mental status examination.

In the adjusted linear mixed-effects model, no significant difference in MMSE trajectory was observed according to baseline living arrangement (p for interaction = 0.55). The estimated annual decline was 0.33 points [95%CI: 0.28; −0.38] overall, 0.28 points [95%CI: 0.19; −0.37] among individuals living alone and 0.34 points [95%CI: 0.28; −0.40] among those not living alone.

## 4 Discussion

### 4.1 Living alone and dementia risk: interpreting the lack of association

In this 5-year follow-up prospective study of 2,269 individuals, neither living alone nor perceived isolation was significantly associated with dementia risk. While several studies have reported that objective and subjective social isolation may increase dementia risk (Yu et al., 2023; Huang et al., 2023), findings remain heterogenous. Some research suggest sex-specific effects (Joyce et al., 2021), or mediation by depression or reduced stimulation (Cardona and Andrés, 2023), while recent findings challenge the protective role or factors like marital status (Karakose et al., 2025).

The absence of association in our study may stem from the relatively healthy, well-educated sample for older people (41% with postsecondary education), which may provide a cognitive reserve that buffers the effects of isolation. Furthermore, our measures may not have fully captured the nuances of social engagement, as living alone or reporting limited closeness does not inherently imply social disconnectedness or a lack of cognitive stimulation.

### 4.2 Institutionalization risk: between social frailty and pragmatic constraints

The principal contribution of this study is not the confirmation that living alone predicts institutionalization but rather the dissociation observed between this outcome and dementia risk. In a cohort specifically constituted of individuals with cognitive complaints or MCI, living alone was not associated with dementia onset or with differential cognitive decline over 5 years. This challenges the assumption that living alone constitutes a uniform risk marker across cognitive ageing outcomes, and suggests instead that its predictive value is specific to pathways of practical and social vulnerability. Previous studies also showed that living alone predicts admission to long-term care facilities, even without cognitive or functional decline (Pimouguet et al., 2016; Takeuchi et al., 2018). A likely explanation is that family usually provides personal assistance, so living alone limits support and increases institutionalization risk. This supports the role of social and environmental contexts in such decisions. Living alone may therefore reflect a broader dimension of social frailty, as suggested by meta-analytic evidence (Kojima et al., 2020). While this association could be viewed through the lens of social frailty, a vulnerability due to insufficient social resources supporting health and wellbeing (Montayre et al., 2024), it is important to consider other explanations.

Indeed, the increased risk of institutionalization likely reflects the practical challenges of maintaining a home without a co-resident caregiver. For individuals with MCI, even in the absence of clinical deterioration, the lack of immediate, “on-site” informal support may make home-based care unsustainable. Family members may opt for institutional care due to geographic distance or the inability to provide the 24-h monitoring sometimes required as difficulties evolve. Thus, living alone may be interpreted less as a marker of biological risk for cognitive decline than as a marker of limited day-to-day environmental support, although this interpretation remains hypothetical.

### 4.3 Study strengths and limitations

To our knowledge, this is the first prospective study to examine both objective and subjective proxys of social isolation in relation to cognitive and institutional outcomes over a 5-year period, using a large national memory clinic-based cohort and longitudinal modeling of cognitive trajectories. The richness of our dataset, the extended follow-up, and the ability to account for a wide range of potential confounders represent key strengths.

However, several limitations must be acknowledged. First, there is a possible selection bias, as participants were recruited from memory clinics and were relatively healthy and highly educated, limiting generalizability to older people with cognitive complaints. Secondly, while our measures distinguished objective (living alone) and perceived isolation, they may not fully capture the complexity of social connectedness and support. Indeed, for the assessment of perceived isolation, we used a single-item self-reported question, which may have led to an underestimation of the prevalence of loneliness, as reflected by the low rate of 6.5% in our cohort compared to the approximately 11% reported in France among individuals aged 60 to 80 (Hansen and Slagsvold, 2016). While we cannot fully exclude that pre-enrolment cognitive changes may have influenced living arrangements prior to study inclusion, the baseline cognitive profile of participants living alone was, if anything, slightly more favourable than that of those not living alone—with identical MMSE scores and a lower proportion of CDR 0.5 — arguing against a major reverse causality effect. Despite these limitations, the stability of cognitive trajectories alongside the increased risk of institutionalization suggests that living alone serves as a marker of practical vulnerability in care support, warranting greater clinical and public health attention to ensure these individuals receive the necessary resources to maintain independent living.

### 4.4 Clinical and public health implications

Beyond epidemiological outcomes, our findings offer insight into the lived experience of individuals with cognitive complaints or MCI. Many older adults wish to remain at home to preserve autonomy. Our results suggest that, for relatively healthy and well-educated adults, living alone was not associated with faster cognitive decline over 5 years. However, the higher institutionalization risk among them underscores the need for strategies to identify and better support those living alone. Enhancing informal networks, and improving access to home-based support services could promote aging at home and help prevent avoidable institutionalization for cognitively vulnerable but functionally preserved individuals.

### 4.5 Conclusion

In conclusion, in this memory clinic-based cohort, neither living alone nor perceived isolation was associated with incident dementia over 5 years. By contrast, living alone was associated with a higher risk of institutionalization, suggesting that it may capture a form of social and functional vulnerability not reflected by dementia incidence alone. Although this finding requires cautious interpretation and replication, it highlights the potential value of living arrangement as a simple clinical marker for identifying cognitively vulnerable older adults who may need earlier support to remain in the community.

## Data Availability

The data analyzed in this study is subject to the following licenses/restrictions: Access to the Memento data is governed by a Data Access Charter that outlines the different steps required throughout the project lifecycle. Each project application is reviewed by the Memento Data Access Committee. Researchers wishing to conduct a study using Memento data may either submit a completed Data Access Charter to sophie.lamarque@u-bordeaux.fr or apply directly online through the Dementias Platform UK (DPUK). Data analysis is conducted via the DPUK portal. DPUK is a UK-based data access platform providing secure access to data from more than 47 dementia-related cohorts. The platform is funded by the Medical Research Council through a public–private partnership and offers a very high level of data security in compliance with the European General Data Protection Regulation (GDPR). Access to Memento data is granted following approval by the Data Access Committee, based on the scientific objectives of the project and the relevance and format of the requested variables. Statistical analyses are performed remotely through a secure web-based connection. Requests to access these datasets should be directed to sophie.lamarque@u-bordeaux.fr and http://www.memento-cohort.org/.
